# Alternative linker histone permits fast paced nuclear divisions in early *Drosophila* embryo

**DOI:** 10.1093/nar/gkaa624

**Published:** 2020-07-25

**Authors:** László Henn, Anikó Szabó, László Imre, Ádám Román, Andrea Ábrahám, Balázs Vedelek, Péter Nánási, Imre M Boros

**Affiliations:** Institute of Biochemistry, Biological Research Centre of Szeged, Szeged H-6726, Hungary; Institute of Biochemistry, Biological Research Centre of Szeged, Szeged H-6726, Hungary; Department of Biochemistry and Molecular Biology, Faculty of Science and Informatics, University of Szeged, Szeged H-6726, Hungary; Doctoral School in Biology, Faculty of Science and Informatics, University of Szeged, Szeged H-6726, Hungary; Department of Biophysics and Cell Biology, University of Debrecen, Debrecen H-4032 Hungary; Department of Biochemistry and Molecular Biology, Faculty of Science and Informatics, University of Szeged, Szeged H-6726, Hungary; Institute of Biochemistry, Biological Research Centre of Szeged, Szeged H-6726, Hungary; Department of Biochemistry and Molecular Biology, Faculty of Science and Informatics, University of Szeged, Szeged H-6726, Hungary; Doctoral School in Biology, Faculty of Science and Informatics, University of Szeged, Szeged H-6726, Hungary; Department of Biochemistry and Molecular Biology, Faculty of Science and Informatics, University of Szeged, Szeged H-6726, Hungary; Department of Biophysics and Cell Biology, University of Debrecen, Debrecen H-4032 Hungary; Institute of Biochemistry, Biological Research Centre of Szeged, Szeged H-6726, Hungary; Department of Biochemistry and Molecular Biology, Faculty of Science and Informatics, University of Szeged, Szeged H-6726, Hungary

## Abstract

In most animals, the start of embryogenesis requires specific histones. In *Drosophila* linker histone variant BigH1 is present in early embryos. To uncover the specific role of this alternative linker histone at early embryogenesis, we established fly lines in which domains of BigH1 have been replaced partially or completely with that of H1. Analysis of the resulting *Drosophila* lines revealed that at normal temperature somatic H1 can substitute the alternative linker histone, but at low temperature the globular and C-terminal domains of BigH1 are essential for embryogenesis. In the presence of BigH1 nucleosome stability increases and core histone incorporation into nucleosomes is more rapid, while nucleosome spacing is unchanged. Chromatin formation in the presence of BigH1 permits the fast-paced nuclear divisions of the early embryo. We propose a model which explains how this specific linker histone ensures the rapid nucleosome reassembly required during quick replication cycles at the start of embryogenesis.

## INTRODUCTION

In eukaryotic cells, genomic DNA is arranged in a highly ordered chromatin structure consisting of nucleosomes as its basic units. Nucleosomes are formed by an octamer core of small, positively charged histone proteins (each composed of dimers of H2A, H2, H3 and H4) wrapped around nearly twice by 145–147 bp DNA. A fifth histone protein, linker histone H1 seals the nucleosome by binding to the entry and exit site of DNA, thus stabilizing a structure called the chromatosome.

Linker histones possess the typical tripartite structure (1) of histone proteins. The central domain has a globular structure, while the lysine-rich N- and C-terminal regions are intrinsically disordered. The globular domain interacts with the nucleosome and the entering/exiting DNA. The C-terminal end is also involved in DNA binding (1–3), while the N-terminus may contribute to the binding affinity (4).

In addition to canonical core and linker histones, which are encoded by high copy number genes, several other histone variants have been identified across metazoans. These variants are known or believed to serve specific functions. Histone variants are transcribed from unique genes in a replication-dependent or –independent manner and in time- and tissue-specific expressional patterns. In humans, 11 linker histone variants are known; seven of these are specific to somatic tissues, while three are expressed exclusively in testis and one in oocyte. Germline-specific variants are typically less conserved than their somatic counterparts, exhibiting differences both in size and amino acid composition.

Although linker histone function is essential in most species, a great deal of functional redundancy is observed among variants, as deletion of specific linker histones frequently results in no obvious phenotype changes. In mice, for example, deletion of either of the somatic H1^0^, H1c, H1d or H1e histone variants does not affect normal development, and neither does H1c/H1e double-mutation. However, H1c/H1d/H1e triple-mutant animals die during embryonic development (5,6).

In general, a specific alternative H1 histone, an oocyte- or early embryo-specific variant is required during the initiation of embryonic development. These H1 variants, maternally deposited into the oocyte, represent the majority of linker histone pool in the early embryo and are later replaced with somatic H1 at the time of zygotic genome activation. The invertebrate sea urchin possesses an H1 variant called CS H1, which is present in the egg and zygote, and is replaced during development by four other, somatic H1 variants (7). In *Xenopus*, the oocyte-specific H1 variant histone B4 is maternally deposited into the egg and persists in the embryo until gastrulation, after which its expression level declines (8,9). The zebrafish H1M variant is present in pre-gastrula stages, but becomes specific to primordial germ cells during gastrulation (10). The mammalian oocyte-specific linker histone H1foo is involved in early development. Ectopic expression of H1foo in mouse embryonic stem cells has been found to result in continuous expression of pluripotent marker genes, thus preventing differentiation (11).

In 2013, BigH1 was described as a *Drosophila* early embryo-specific functional linker histone variant with an unusual amino acid composition (12). Similar to *Drosophila* canonical H1 (H1), BigH1 also possesses the typical tripartite structure. However, the N-terminal domain of BigH1 is considerably larger and richer in acidic amino acids. The most conserved central globular domain of BigH1 shows only 57% homology to canonical H1. BigH1 is highly abundant in the precellular embryo when nuclei divide with 13 extremely rapid metasynchronous cleavages without cytokinesis. Following the fast replicative period in nuclear cycle 14 (NC 14), nuclei undergo the cellularization process, and consecutive rapid nuclear divisions are replaced by slower mitotic cell cycles. By cellularization BigH1 is switched to H1 in most cells, but is retained in the primordial germ cells at the posterior part of the embryo. The switch also corresponds with the time when the zygotic genome is activated, resulting in a high level of transcripts from the previously compacted, silent embryonic chromatin, and the decline of maternal transcripts which regulated development up until this point (12). A role for BigH1 in preventing early zygotic gene activation was suggested and a recent report demonstrated transcription inhibition by the N-terminal domain of BigH1 in *in vitro* assay of S2 cells (13).

Intrigued by the observations that in most organisms an alternative linker histone is required during the early stages of embryonic development, we were interested to know whether a functional comparison of the canonical H1 and the sole *Drosophila* H1 variant, BigH1 can provide novel information on the specific histone function required at the start of embryogenesis. We took advantage of the unique situation of *Drosophila* possessing a sole H1 variant and approached this question by generating BigH1–H1 hybrid proteins. For these the coding sequence of BigH1 was replaced completely or partially with that of H1, resulting in animals that express H1 or chimera H1/BigH1 histone protein under the control of *BigH1* regulatory sequences. Based on the analysis of these fly lines, we show that while BigH1–H1 chimeras or even H1 can substitute for BigH1 and support embryonic development at optimal temperatures, the globular and C-terminal domains of BigH1 are required for proper nuclear cleavages and development at low temperature. We found that the complete replacement of BigH1 with H1 results in a larger nuclear volume, indicating alteration in chromatin organization. We show that BigH1 facilitates dynamic nucleosome exchange during S-phase in the syncytial blastoderm embryo, thus accommodating the rapid divisions of nuclei as a highly mobile linker histone, while also stabilizing nucleosomal structure. Here we propose a model for describing the interaction between BigH1 and nucleosomes and explaining BigH1 function in early *Drosophila* embryogenesis.

## MATERIALS AND METHODS

### Establishment of mutant fly lines

*BigH1* mutant alleles were generated by CRISPR/Cas9 gene editing. gRNA target sites were identified by CRISPR Optimal Target Finder. gRNA coding sequences (Upstream protospacer sequence: 5′-ATTAGCAGTGTTATTCCATA-3′; downstream protospacer sequence: 5′-ATAATACCTCTAGAAGGAAT-3′) were cloned into pCFD4 plasmid as described by Port *et al.* (14). pCFD4 encoding gRNAs against *BigH1* genomic loci were injected into *y[1] v[1] P{nos-phiC31\int.NLS}X; P{CaryP}attP40* embryos (15) (500 ng/μl plasmid DNA in injection buffer (5 mM KCl, 0.1 mM KH_2_PO_4_, pH 7.8)), to insert transgenic gRNA genes into the 2nd chromosome of the *Drosophila* genome.

**Figure 1.**
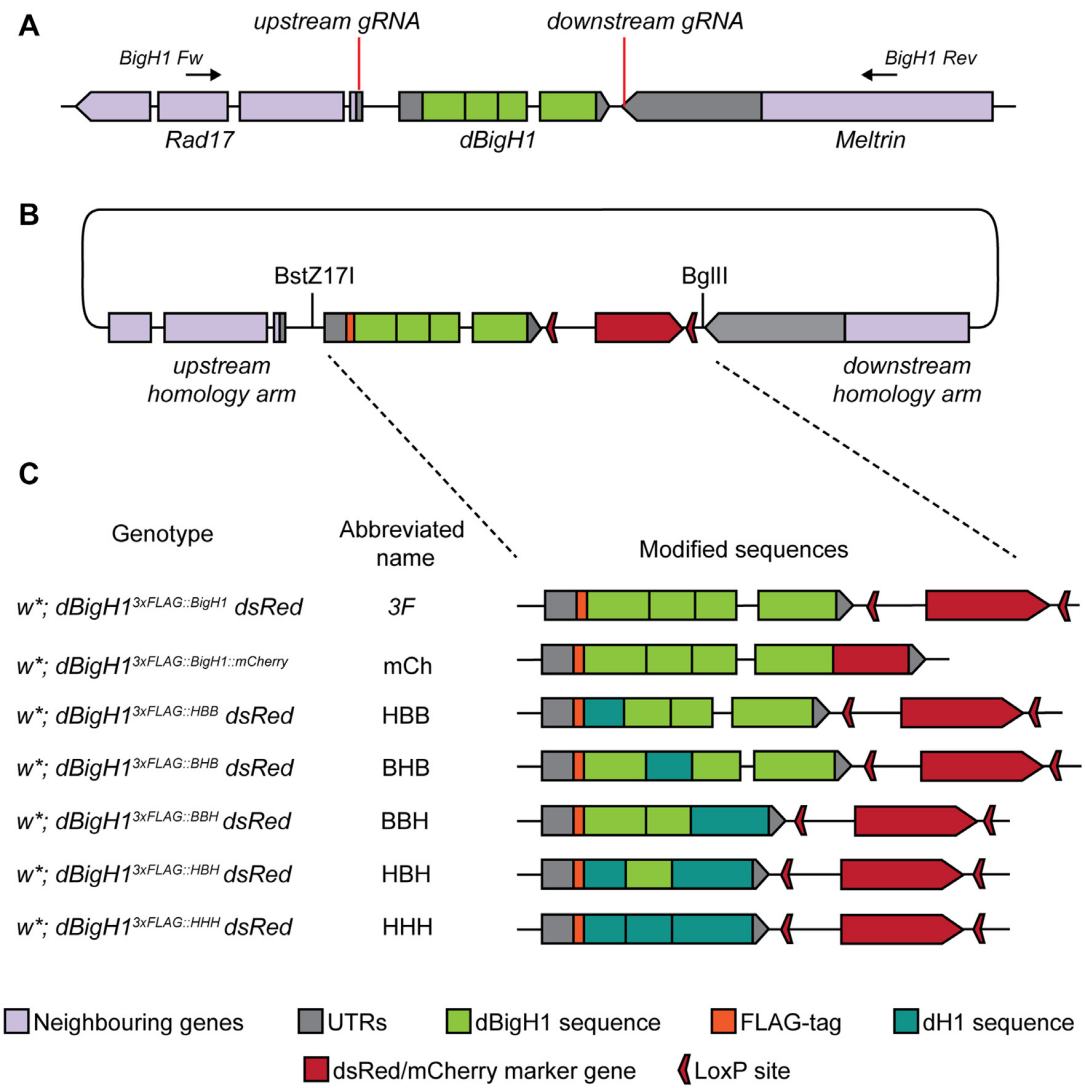
CRISPR/Cas9-guided generation of chimeric BigH1/H1 alleles. (**A**) Schematic view of the *BigH1* genomic region with the indicated target sites of the gRNAs used for CRISPR/Cas9 gene editing. (**B**) Schematic map of the donor plasmid for generating the *3F* allele. The donor plasmid carries upstream and downstream homology arms, *dsRed* marker gene surrounded by LoxP sites and the modified *BigH1* encoding sequence. Manipulated sequences were inserted between BstZ17I and BglII restriction sites. (**C**) Genotypes of BigH1 mutant animals, abbreviated name and general structure of generated *BigH1* alleles.

For the precise modification of the endogenous *BigH1* sequence, we generated donor plasmids by inserting a 4640 bp long genomic fragment amplified with *BigH1 Rev* (5′-ggacacactgacatttagctgtttgg-3′) and *BigH1 Fw* (5′-tactccgtaattgatgagattccgcc-3′) primers into pTZ57R/T plasmid (Thermofischer Scientific). The donor plasmid carries a 3xP3 promoter-driven *dsRed* sequence as a marker gene, 16 bp from *BigH1*’s 3′UTR. PAM sequences were mutated on the donor plasmid by PCR mutagenesis using BigH1 PAM upstream (5′-ACCTTCCGTACGCTCATTTTTAGAATTAACTCCTCTTCTTCTTTAGCAGTGTTAACATATG) and BigH1 PAM downstream (5′-AACTCAATTGAAATGAGAAAATGTGTTTATAATAGCTCTAGAAGGAATAAGCGATAACCGAG) primers. Mutant BigH1 sequences were subcloned in pUC18 plasmid carrying the BstZ17I and BglII fragment (2843 bp) of the donor plasmid (Figure 1B). All modifications of the original *BigH1* sequence were made by Sequence and Ligation Independent Cloning (SLIC) (16). The 3xFLAG epitope encoding sequence was built into all modified genes following the first methionine. For cloning the *BigH1*–*H1* chimera genes, we used the following domain borders: BigH1 N-terminal domain: M1-K97; BigH1 central domain: P98-T168; BigH1 C-terminal domain: D169-STOP353; H1 N-terminal domain: M1-P44; H1 central domain: S45-A119; H1 C-terminal domain: S120-STOP257. After subcloning in pUC18 plasmid, modified *BigH1* sequences were inserted into the donor plasmid by BstZ17I and BglII restriction enzymes, exchanging the wild type *BigH1* sequence.

Donor plasmids encoding modified BigH1 sequences were injected (500ng/μl plasmid DNA in injection buffer) into embryos laid by *y[1] M{vas-Cas9}ZH-2A w^1118^* females (BDSC_51323) crossed with transgenic *BigH1* gRNA expressing males. Gene replacements were identified by *dsRed* expression in male descendants of injected males and verified by Sanger sequencing.

mCherry tagged wild type BigH1 allele (*mCh*) was made similarly, except that in that case the donor plasmid did not carry the 3xP3 promoter driven dsRed marker gene, and gene replacement in the descendants of injected males was identified by PCR using primers specific for mCherry (mCh Fw: 5′-GTGAAGCTGCGCGGCACC) and genomic sequences (BigH1 PAM downstream).

### Protein extract preparation and Western blotting

Protein fractions were prepared from 1–2 and 3–4 h old dechorionated embryos, following the protocol described in Pérez-Montero *et al.* (12) with minor modifications. Frozen embryos were homogenized using a plastic pestle in Buffer A/1 (0.23 M sucrose, 15 mM Tris–HCl pH 7.5, 60 mM KCl, 15 mM NaCl, 0.15 mM spermine, 0.5 mM spermidine, 0.2 mM PMSF, 14 mM mercaptoethanol, 0.25 mM MgCl_2_) and centrifuged at 3300 g for 15 min at 4°C. The supernatant was transferred to a clean tube and used as non-chromatin-bound protein fraction. The pellet was washed once with Buffer A/1 and Buffer A/2 (15 mM Tris–HCl pH 7.5, 60 mM KCl, 15 mM NaCl, 0.15 mM spermine, 0.5 mM spermidine, 0.2 mM PMSF, 14 mM mercaptoethanol, 0.25 mM MgCl_2_) each. For salt elution the pellet was resuspended in Buffer A/2 supplemented with NaCl as indicated and kept on ice for 10 min, then centrifuged at 3300 g for 15 min at 4°C. The supernatant was transferred to a clean tube, as the salt eluted fraction, and the pellet (chromatin fraction) was resuspended in Buffer A/2.

A list of primary and secondary antibodies is shown in Supplementary Table S1. Protein expression levels normalized to H3 loading control were determined using ImageJ software (National Institutes of Health, USA).

### Immunohistochemistry

Embryos were dechorionated with 50% bleach, fixed in 1:1 mixture of heptane:PBS and 4% formaldehyde for 20 min, devitellinized with 1:1 heptane:methanol mixture and stored in methanol. Embryos were rehydrated by washing with 2:1, 1:1 and 1:2 mixture of methanol:PBT (0,1% Triton X-100 in 1× PBS) for 3 × 5 min, then washed in PBT for 3 × 5 min and blocked in PBT-N (0.1% Triton X-100, 1% bovine serum albumin, 5% fetal calf serum in PBS) at room temperature for 1 h. Samples were incubated with primary antibody diluted in PBT-N overnight at 4°C, washed with PBT for 3 × 15 min at room temperature, then incubated with the secondary antibody and DAPI. Embryos were washed with PBT for 3 × 10 min and placed on microscope slides and mounted in Fluoromount-G (Invitrogen^®^). A list of primary and secondary antibodies is shown in Supplementary Table S1.

For the evaluation of nuclear fallout phenotype, embryos were visualized with spinning disk confocal microscope (Visitron spinning disk confocal microscope with Yokogawa CSU-W1 unit and Andor Zyla 4.2 PLUS sCMOS camera) using 20× dry objective (NA: 0.45). 2 μm optical sections were captured at the midsagittal plane of embryos (4 × 0.5 μm Z-stacks). Nuclei were counted manually in the dorsal or lateral cortical layer. For each genotype 20–37 embryos were analyzed, permitting evaluation of 60 nuclei in each on average. For statistics of nuclear fallout phenotype two-tailed unpaired *t*-test was used. Confocal images of nuclear fallout phenotype and other immunostainings were made with Leica SP5 AOBS confocal laser scanning microscope with 20× dry (NA: 0.7) objective. Composite images were prepared using ImageJ software.

### Determination of embryo viability at different temperatures

Wild type and homozygous mutant flies were kept at 25°C. 0–30 min old embryos were collected and placed at 25, 30 and 15°C, and hatching rates were determined after 36 or 96 h (in the case of 15°C) after egg laying by counting hatched and unhatched embryos. For statistics of embryo viability two-tailed, unpaired *t*-test was used.

### MNase assay for chromatin accessibility

Chromatin was prepared from 0.5 to 2.5 h dechorionated embryos, following the protocol described in (12) with some modifications. Frozen embryos were homogenized using a plastic pestle in Buffer A/1 (0.23 M sucrose, 15 mM Tris–HCl pH 7.5, 60 mM KCl, 15 mM NaCl, 0.15 mM spermine, 0.5 mM spermidine, 0.2 mM PMSF, 14 mM mercaptoethanol, 0.25 mM MgCl_2_) and centrifuged at 3300 g for 15 min at 4°C. The pellet was washed once with Buffer A/1 and Buffer A/2 (15 mM Tris–HCl pH 7.5, 60 mM KCl, 15 mM NaCl, 0.15 mM spermine, 0.5 mM spermidine, 0.2 mM PMSF, 14 mM mercaptoethanol, 0.25 mM MgCl_2,_ 1 mM CaCl_2_) each and resuspended in Buffer A/2. The concentration of the obtained chromatin preparation was determined using Nanodrop 2000. For MNase (Thermofisher Scientific) digestion 240 ug chromatin was incubated with 2 U of enzyme in Buffer B (10 mM Tris–HCl pH 7.5, 60 mM KCl, 15 mM NaCl, 0.15 mM spermine, 0.5 mM spermidine, 1 mM CaCl_2_) for 30 min at 37°C. Following digestion, EDTA was added to 10 mM final concentration and samples were kept on ice for 5 min. DNA was further purified by RNase digestion (50 ug/ml, at 37°C, 20 min) and protein denaturation by 1% SDS, 1 M NaCl, followed by phenol:chloroform extraction. The obtained DNA was analyzed using a Bioanalyzer 2100 (Agilent) DNA 1000 chip. For statistics two-tailed, unpaired *t*-test was used.

### Live imaging of *Drosophila* embryos and FRAP measurements

*H2Av-GFP; +* and *H2Av-GFP; HHH* homozygous embryos were collected 0–1 h after egg laying, dechorionated with 50% bleach and washed with water, then aligned on glue glass coverslips and covered with halocarbon oil. Live imaging was performed using spinning disk microscope under 10× dry (NA: 0.3) or 100× oil immersion (NA: 1.3) objectives. Embryos were recorded for 2 h with 6 frame/min or 20 frame/min. In nuclear cycle length measurement analysis, 27 and 25 embryos of *H2Av-GFP; +* and *H2Av-GFP; HHH* genotypes were recorded, respectively.

Nuclear cycle lengths and nuclei number were determined using a 20× dry objective with 6 frame/min time resolution in 25 and 24 of *H2Av-GFP; +* and *H2Av-GFP; HHH Drosophila* embryos, respectively.

Nuclear diameters of NC 10–13 embryos were determined in S-phase ∼2 min after mitosis, when nuclei have the largest size, using spinning disk microscope with a 100× oil immersion objective. For each genotype 21–60 nuclei of three embryos were measured and nuclear volumes were calculated. Histone carrying lipid droplet densities were also determined on these movies. LDs were counted manually in 150 × 150 pixel (97.38 μm^2^) areas. 12–12 measurements were made at early S-phase of NC 10–12 in both genotypes. For statistics of nuclear cycle lengths, number of nuclei, nuclear diameter and LD densities two-tailed, unpaired *t*-test was used.

FRAP experiments were performed using Leica SP5 AOBS confocal laser scanning microscope with a 40× dry objective (NA: 0.75) and Leica FRAP Wizard. The fluorescence recovery of the GFP signal originating from H2Av-GFP core histone expressed from transgene (BDSC_24163) was monitored in NC 11 and 12. Both for wild type and *HHH* mutant embryos 12–12 nuclei in early (approx. 20–150 s after mitosis) and 10–11 nuclei in late (∼150–300 s after mitosis) S-phase were investigated in FRAP experiments. Recordings in FRAPs included prebleach 1×, bleach: 4 × 200 ms (time interval 1.314 s), postbleach 20× (time interval: 5 s). Since nuclei were moving because of cytoplasm drifting, fluorescence recovery was measured manually frame by frame using ImageJ software (National Institutes of Health, USA). Datasets were normalized using the easyFrap web tool. As the short time intervals of measurements were not sufficient to determine FRAP parameters such as *t*_1/2_ and mobile/immobile fraction precisely, area under curve of individual measurements during the first 40 s were used for statistical analysis, since nucleosome exchange is the most dynamic during this interval. Statistical analysis (two-tailed, unpaired *t*-test) was performed and results were visualized by GraphPad Prism6 software (version 5.00 for Windows, San Diego, CA, USA) using non-linear one phase association, and SEM as error bar.

### Nucleosome stability assay

Frozen embryos were homogenized with a plastic pestle in Buffer I/A (300 mM sucrose, 10 mM HEPES pH 7.9, 10 mM KCl, 1.5 mM MgCl_2_, 0.1 mM EGTA, 0.5 mM DTT, 0.5 mM PMSF, 1× protease inhibitor cocktail) and centrifuged at 550 g for 1 min at 4°C. The supernatant was transferred to a clean 1.5 ml tube and centrifuged at 1300 g for 10 min at 4°C. The pelleted nuclei were resuspended in Buffer I/A. Equal volume of Buffer I/B (1,7 M sucrose, 10 mM HEPES pH 7.9, 10 mM KCl, 1.5 mM MgCl_2_, 0.1 mM EGTA, 0.5 mM DTT, 0.5 mM PMSF, 1× protease inhibitor cocktail) was loaded in a clean tube and the sample pipetted on top of the sucrose cushion. The boundary layer was gently mixed, and samples were centrifuged at 28 000g for 15 min at 4°C. The pellet was resuspended in Buffer II (10 mM HEPES pH 7.9, 10 mM KCl, 2 mM MgCl_2_, 0.5 mM DTT, 0.5 mM PMSF, 1× protease inhibitor cocktail). Nuclei were embedded in glass bottom ibidi^®^ eight-well plates as described (17). After propidium-iodide staining and wash steps, nuclei were visualized using a Spinning Disc confocal microscope with a 40× objective (NA: 0.6). For image analysis iCys 7.0 software for Windows 7 was used. Data normalization and evaluation was performed as described in Imre *et al.* (17).

### Structural comparison of BigH1 and dH1 globular domains

BigH1 (CG3509), dH1 (CG31617) and chicken H1 (P02259) sequences were obtained from public databases. Globular domain sequences of the above histones (BigH1 97–169, dH1 45–119, gH5 24–98) were aligned with ClustalW algorithm (gap opening penalty 15, extension 1.2) (18). Nucleosome structures with linker histone (5NL0, 5WCU, 4QLC) were downloaded from PDB and compared with UCSF Chimera (19). Structures of dH1 and BigH1 sequences were modelled by Modeller (20) using the gH5 linker histone structure (4QLC) as template. Structure comparisons were made using UCSF Chimera including identification of amino acids described in DNA binding by Zhou *et al.* (21). Further model prediction was performed using Phyre2 (22).

## RESULTS

### BigH1-H1 chimeras can substitute BigH1 in early embryonic chromatin

For the analysis of the specific function of *Drosophila* alternative linker histone BigH1 during early embryogenesis, we used CRISPR/Cas9 mediated homologous recombination to generate its mutant alleles, producing chimera proteins (Figure 1). During the construction of these alleles only protein encoding sequences were manipulated; UTRs and surrounding sequences were not altered. Gene replacements via homologous recombination were achieved by injection of donor plasmids carrying modified *BigH1* sequences between homology arms into embryos expressing gRNAs and *vasa* promoter-driven Cas9 protein (Figure 1A and B). An mCherry-tagged wild type BigH1 sequence was also generated to allow the visualization of BigH1 expression pattern in live embryos. In one construct the entire BigH1 coding sequence was replaced with that of H1 (*HHH*). Four further alleles encode chimera BigH1/H1 proteins, consisting of BigH1 and H1 domains (the designations of these alleles indicate their domain composition: first letter refers to N-terminal, second to central, and third to C-terminal domain coding H1 or BigH1 sequences (Figure 1C). To facilitate identification and detection of each of the proteins expressed from new *BigH1* alleles, they were constructed to have a 3× FLAG epitope at the N-terminus. To verify that this modification does not cause changes in the expression pattern or function of the protein, a 3× FLAG-tagged wild type *BigH1* allele (*3F*) was also generated.

**Figure 2.**
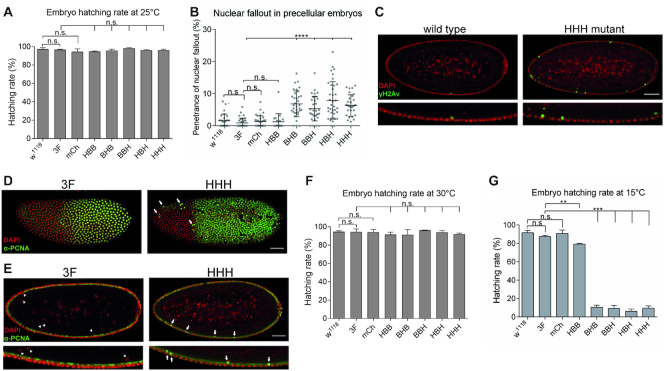
*BigH1* mutant embryos showed nuclear fallout phenotype and cold sensitivity. (**A**) Hatching rate of wild type and *BigH1* mutant embryos at 25°C. (**B**) Nuclear fallout phenotype observed in wild type and BigH1 mutant embryos. (**C**) Immunostaining of typical wild type and *HHH* mutant embyos representing nuclear fallout phenotype. (**D**) PCNA staining of *3F* and *HHH* syncytial blastoderm embryos during a mitotic wave. Arrows indicate nuclei in which the nuclear cycle cannot progress to mitosis. (**E**) Nuclear fallout in *3F* and *HHH* syncytial blastoderm embryos. Eliminated nuclei do not show PCNA staining in *3F* embryos (arrowheads), while in *HHH* mutant embryos descending nuclei are PCNA positive (arrows). Nuclei in the cortical layer are in non-replicative phase in both embryos, indicated by negative PCNA staining. Scale bar represent 50 μm (C–E). (**F–G**) Hatching rate of wild type and *BigH1* mutant embryos at 30 and 15°C, respectively. For statistics, two-tailed, unpaired *t*-test was used, error bars represent SD, *P*-values: **<0.01; ***<0.001 (A, B, F, G).

Embryos homozygous for any of the constructed new alleles were fully viable and fertile under normal conditions. There was no significant difference in the viability of embryos laid by homozygous mutant females at 25°C, indicating that any domain or even the entire protein coding sequence of *BigH1* can be replaced by the corresponding sequences of *H1* (Figure 2A). Live imaging of *mCh* syncytial blastoderm embryos shows that mCherry-tagged BigH1 protein is evenly localized in the nuclei in S-phase and on the chromosomes throughout mitosis, indicating that the modified protein behaves as expected for a linker histone (Supplementary movie S1). To assess whether the generated new alleles encode functional proteins which show similar distribution to wild type BigH1, we performed immunostaining experiments using anti-FLAG antibody. FLAG epitope-tagged proteins expressed from BigH1 alleles localized in the nuclei of syncytial blastoderm embryos. During cellularization, staining became weaker in somatic cells, but strong signal remained in pole cells. In later stages, staining was preserved in the primordial germ cells only (Supplementary Figure S1). Together, these observations suggest that proteins expressed from modified BigH1 genes behave as the wild type BigH1 protein (12). Immunostaining of embryos by anti-FLAG antibody allowed the comparison of protein amounts expressed from the different newly generated alleles. Interestingly, despite the finding that embryos homozygous for either modified BigH1 allele showed similar progress in development to wild type, immunostaining showed that the FLAG staining was weaker in mutants where BigH1’s N-terminal domain was substituted with that of H1 (Supplementary Figure S1). Western blots of proteins extracted from embryos showed that indeed the protein levels of HBH, HBB and HHH chimeras were indeed lower than the level of wild type BigH1, in accord with the result of immunostaining experiments (Supplementary Figure S2). This shows that the level of protein expressed from a gene carrying the coding region that corresponds to the N-terminal domain of H1 instead of that of BigH1 is reduced in embryos, despite every other coding and regulatory sequence and the chromosomal position of the gene being identical to that of BigH1. The mechanism resulting in this altered expression level is so far unknown.

### The globular and C-terminal domains of BigH1 are required for its proper function

In early syncytial blastoderm *Drosophila* embryos, cycles of extremely rapid nuclear cleavages follow each other without effective DNA damage control. During this developmental stage, damaged nuclei are eliminated from the proliferating nuclear layer by a Chk2-dependent mechanism (23). Descent of damaged nuclei from the cortical nuclear layer into the underlying yolk is a well observable phenomenon called nuclear fallout. To investigate whether BigH1 mutant embryos show abnormalities during precellular development, we visualized nuclear fallout phenotype by immunostaining.

We observed that despite the full viability of *BigH1* mutant embryos, in mutants where the globular or C-terminal domain of BigH1 was changed to H1 sequences 5.4–7.9% of the nuclei showed nuclear fallout on average, while in wild type, *3F* and *mCh* embryos these values ranged between 0.9 and 1.3% (Figure 2D and E). Descending nuclei were positive for γH2Av staining, a hallmark of double stranded DNA breaks (24), indicating that this phenotype is caused by DNA damage. The nuclei eliminated through nuclear fallout were also positive for PCNA (Figure 2F and G) indicating that nuclear fallout is a consequence of double-stranded DNA breaks resulting from improper replication. The elevated ratio of improper divisions in the syncytial blastoderm stage of these embryos suggests that the central globular and C-terminal domains of BigH1 are required for proper nuclear cleavages in syncytial embryos. The penetrance of this phenotype, however, is rather low at normal temperature, indicating that the replacement of these domains with those of H1 does not cause serious mitotic defects. Interestingly, changing the most different N-terminal domain between BigH1 and H1 does not cause the increase of nuclear fallout phenotype.

Although BigH1 mutants did not display obvious change in embryo viability under normal conditions, we asked whether the same was also true under suboptimal temperatures. For this purpose, we measured the embryo hatching rate at 15 and 30°C. Since egg production and male fertility decrease at low and high temperatures, respectively, we kept flies at 25°C and transferred embryos to 15°C or 30°C. Development at high temperature had no effect on the phenotype (Figure 2B). However, at low temperature embryo viability was strongly reduced in mutants in which the C-terminal or/and globular domains were H1 type (*BHB*, *BBH*, *HBH*, *HHH*), compared to the wild type BigH1 (Figure 2C). Interestingly, in the *HBB* mutant, where only the N-terminal domain was replaced, we measured only a weak decrease in hatching rate at lower temperature, despite the fact that these embryos had lower linker histone protein level. The lethality of mutants is consistent with their observed nuclear fallout phenotypes, indicating the importance of the central and C-terminal domains of BigH1 for proper pre-cellular nuclear cleavages. *3F* and *mCh* mutants showed normal nuclear cycles and similar viability to wild type, indicating that neither genome manipulation by CRISPR/Cas9 (insertion of dsRed marker gene or modification of PAM sequences), nor the FLAG epitope, nor mCherry fluorescent tag influenced this phenotype.

Next, to find an explanation for the cold sensitive phenotype, we further analyzed *HHH* mutant embryos as a representative of the type showing lethality. Immunostaining experiments revealed strong mitotic defects in these embryos observable as aggregated DNA (Supplementary Figure S3, middle panel) and loss of synchronized divisions (Supplementary Figure S3 bottom panel) in early, but not late embryonic stage. In several cases, in *HHH* mutant embryos only a subset of precellular nuclei survived in islets until NC 10–11 (nuclei were localized in the cortex and showed elongating RNA polymerase II staining), and the majority of nuclei were lost (Supplementary Figure S3A, bottom panel), suggesting that very early NCs were affected as well, and the effect of nuclear damage appeared in a clonal manner. These results demonstrate that BigH1 provides function(s) that H1 cannot substitute at low temperature.

### Chromatin structure on nucleosome level is similar in wild type and *HHH* mutant

Next, we focused on exploring phenotypic differences of wild type and *HHH* mutant embryos at optimal temperature (25°C) in order to gather information on the specific function of BigH1 in the early embryo. For this we used *H2Av-GFP* transgene as genetic background to visualize changes in chromatin structure during nuclear divisions. Comparisons of wild type and *HHH* mutant syncytial blastoderm embryos by live imaging revealed higher number of nuclei removed from the cortical layer in the *HHH* mutant, resulting in lower number of nuclei compared to the wild type (Supplementary Figure S4A). As a consequence, *HHH* mutant embryos often showed extra precellular cleavages (5 of 24 embryos) compared to the wild type (1 of 25 embryos) (Supplementary movie S2). However, the lengths of individual nuclear cycles were not altered significantly in mutant embryos as compared to wild type ones (Supplementary Figure S4B).

**Figure 3.**
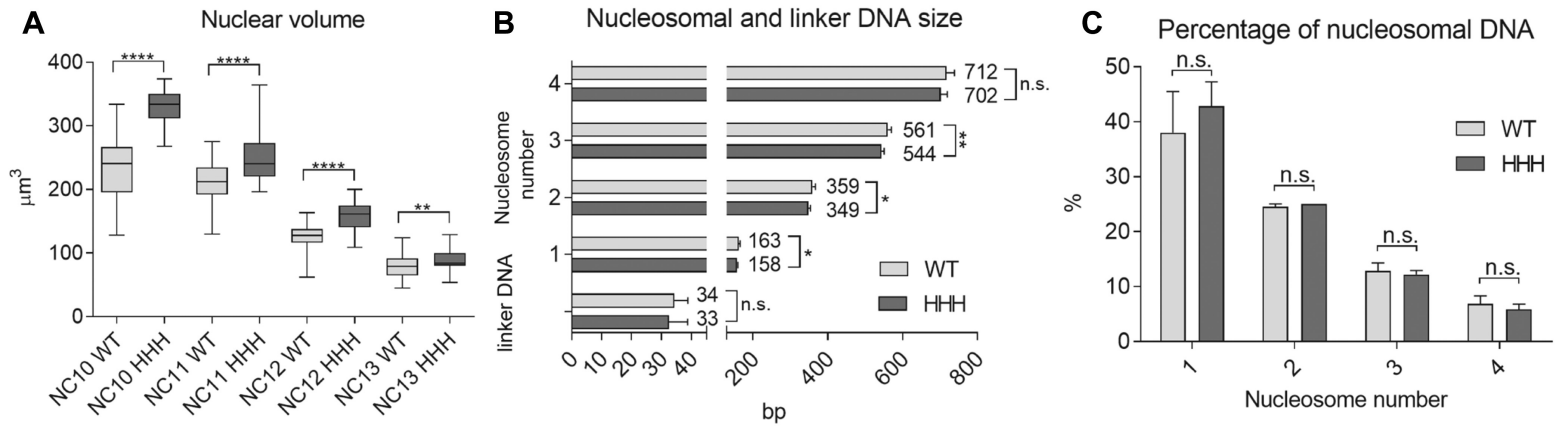
Wild type and *HHH* mutant embryos differ greatly in nuclear volume but only slightly in nucleosomal DNA length. (**A**) Nuclear volume at the nuclear cycles leading up to cellularization (NC 10–13). The diameters of nuclei were measured in H2Av-GFP expressing precellular embryos, and their volume calculated. (**B**) Mono- and multinucleosomal DNA size and linker DNA length compared in WT and HHH mutant, determined by running MNase digested sample DNA on a Bioanalyzer DNA 1000 chip. (**C**) Percentage of mono- and multinucleosomal DNA in wild type and *HHH* mutant embryonic chromatin, to assess the general compaction state of chromatin. For statistics, two-tailed, unpaired *t*-test was used. Error bars represent SD, *P*-values: *<0.05; **<0.01; ****<0.0001 (A–C).

We also observed a significant difference in the size of nuclei between *HHH* mutant and wild type syncytial embryos. We measured the diameter and calculated the volume of nuclei in NC 10–13, and found that nuclei were consistently bigger in *HHH* embryos. The difference was observable throughout NC 10–13, however, it was most prominent in NC 10 and 11 (Figure 3A). Our hypothesis for the differing volume was that chromatin structure of the *HHH* mutants is less compacted than it is in wild type animals, thus they show an expansion of nuclear volume. To test this, we designed MNase digestion assay to assess nucleosomal DNA size, linker DNA length and the ratio of mononucleosomal DNA (indicative of the ratio of open and closed chromatin). Digested chromatin samples were run on Bioanalyzer to get base pair resolution of DNA size. We found that while linker DNA length did not change between *HHH* and wild type nuclei, mono- and multi nucleosomal DNA size was slightly shorter in the *HHH* mutant (Figure 3B). The ratio of mononucleosomal to multinucleosomal DNA was also similar in wild type and mutant chromatin (Figure 3C). Thus, the difference in nuclear volume is unlikely to be the result of differences in chromatin structure on nucleosome level. Nonetheless, these results might indicate a difference between H1 and BigH1 binding to nucleosomes and that a larger area of DNA is protected from digestion in BigH1 containing chromatosomes.

### BigH1 facilitates dynamic nucleosome exchange in the early S-phase

**Figure 4.**
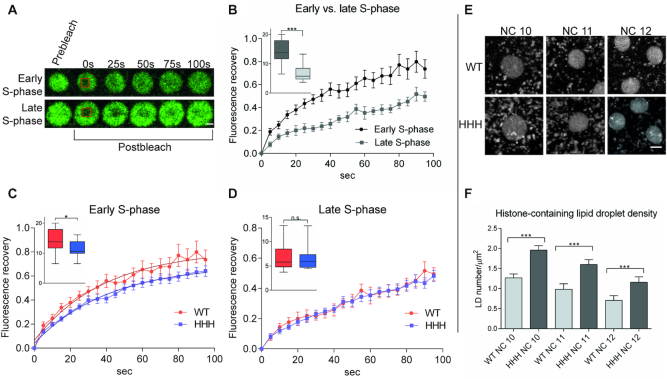
Nucleosome exchange is less dynamic in chromatin formed in the presence of H1 in early embryonic nuclei (**A**) Images of early and late S-phase in nuclei of *H2Av-GFP;+* (wild type) syncytial blastoderm embryos before and after photobleaching. Scale bar represents 2 μm. Recovery of fluorescent signal originating from transgenic H2Av-GFP refers to nucleosome dynamics. (**B**) Nucleosome dynamics in early and late S-phase in wild type embryos. Comparison of nucleosome dynamics in wild type and *HHH* mutant embryos in (**C**) early and (**D**) late S-phase. Error bar: SEM. Box plots on these diagrams show values of area under curve of individual FRAP measurements in the first 40 seconds where error bars represent SD. For statistics, two-tailed, unpaired t-test was used (B–D). (**E**) Images of embryonic nuclei of *H2Av-GFP; +* (wild type) and *H2Av-GFP; HHH* embryos in the early S-phase of NC 10, 11 and 12 display higher number of H2Av-GFP containing LDs in *HHH* mutant embryos. Scale bar represents 2 μm. (**F**) Quantitation LD density in wild type and *HHH* mutant embryos in different stages. Error bars represent SD. For statistics, two-tailed, unpaired *t*-test was used, *P* values: *<0.05, ***<0.001 (B–D, F).

As obvious differences in the nucleosomal structure between the chromatin of *HHH* and wild type embryos do not seem to offer an explanation for the observed differences in phenotypes, we tested if alterations in the dynamics of chromatin structural changes during the rapid stages of nuclear cycles can be found. To investigate this, we performed FRAP experiment to determine the fluorescence recovery of H2Av-GFP signal after photobleaching in the nuclei of *H2Av-GFP;+* and *H2Av-GFP; HHH* precellular embryos. Since core histones are tightly bound to DNA, short period FRAP studies (from several seconds to few minutes) on GFP tagged core histones usually show a highly immobile fraction and slow recovery time (25). We investigated H2Av dynamics by FRAP at the S-phase of pre-cellular nuclei of wild type and *HHH* embryos, expecting rapid core histone exchange during the replication of DNA. We observed that fluorescence recovery of the H2A-GFP signal differed in early and late S-phase. In early S-phase (20–150 s after mitosis) a more mobile fraction was detected compared to late S-phase (150–300 s after mitosis), suggesting that nucleosome dynamics strongly differ at these two stages (Figure 4A and B). Therefore, we compared nucleosome exchange in wild type and *HHH* embryos both in early and late S-phase. In early S-phase, an obvious difference was observed between wild type and *HHH* mutant embryos, since fluorescence showed faster recovery in the bleached area in wild type, suggesting that BigH1 facilitates a more dynamic nucleosome exchange than H1 during replication (Figure 4C). This difference is absent in late S-phase when most of replication and, with it, nucleosome exchange has already happened (Figure 4D). Taken together, these results revealed that BigH1 allows a more dynamic nucleosome exchange than somatic linker histone H1 during the rapid replications in precellular *Drosophila* embryonic nuclei.

Another observation we made by *in vivo* analysis of nuclear divisions of H2Av-GFP carrier wild type and *HHH* mutant embryos also supports the notion of altered chromatin dynamics. In the syncytial blastoderm embryo, maternally provided core histone proteins are stored in lipid droplets (LDs) during early embryogenesis (26,27) and are translocated from LDs to nuclei very dynamically (28). Live imaging of *H2Av-GFP;+* and *H2Av-GFP;HHH* embryos revealed that in the *HHH* mutant there are more fast moving GFP positive spots around the nuclei (Figure 4E and F). As the GFP signal comes from the H2Av-GFP transgene, we conclude that these spots correspond to LDs carrying core histones. We assume that the elevated number of LDs in the *HHH* mutant is the consequence of less dynamic nucleosome exchange.

### Nucleosomes formed in the presence of H1 are less stable than those assembled with BigH1

To compare the stability of nucleosomes formed in the presence of BigH1 and H1 more directly, we tested the resistance of different core nucleosomal histones toward salt elution, making use of two different assays. The Quantitative Imaging of Nuclei after Elution with Salt/Intercalators (QINESIn) technique uses nuclei for *in situ* quantitative measurement of nucleosome stability (17). We have optimized this method for embryonic nuclei extracted from whole *Drosophila* embryos. H2Av-GFP containing nuclei were isolated from wild type and *HHH* syncytial blastoderm embryos, embedded into agarose, permeabilized and washed with increasing concentrations of sodium-chloride. After wash steps, nuclei were fixed with formaldehyde and stained with propidium-iodide (PI) to label DNA. Following these steps embedded nuclei were analyzed by confocal microscopy to determine PI and GFP signal intensities. Changes in the ratio of GFP and PI signal intensity indicate elution of H2Av from the chromatin by salt wash, reflecting nucleosome stability.

**Figure 5.**
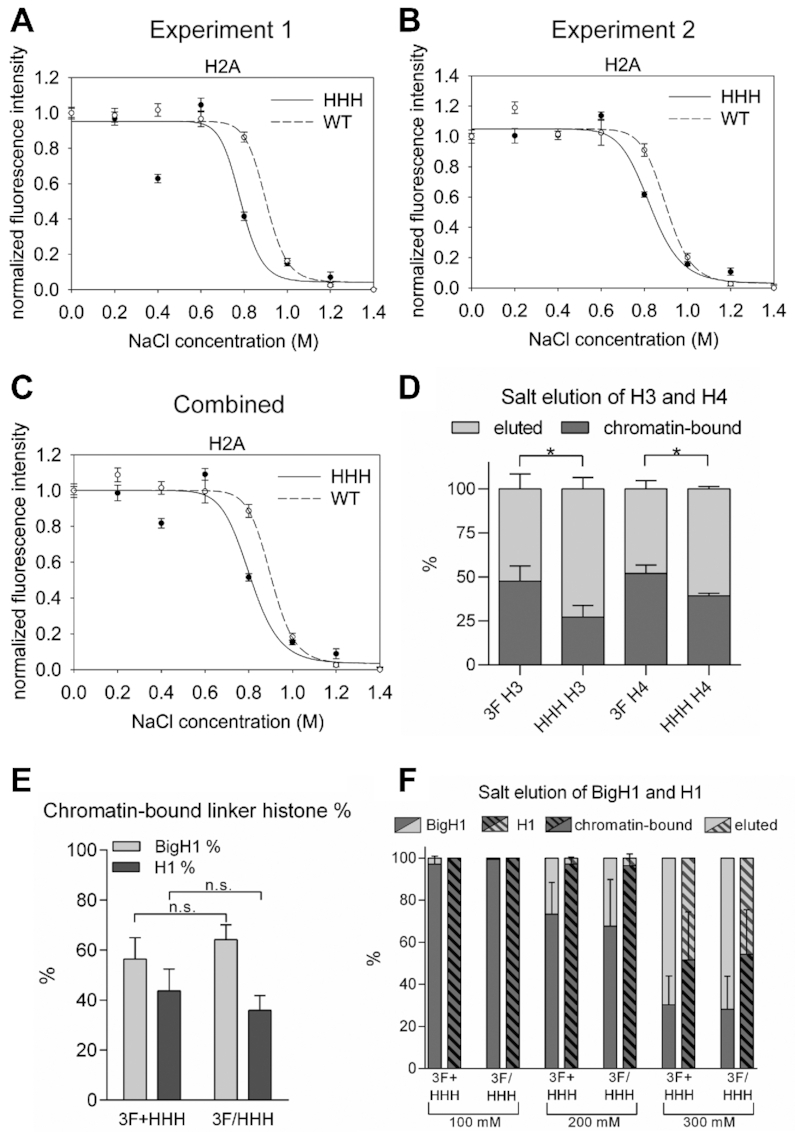
Nucleosomes formed in embryos in the presence of H1 are less stable. (**A, B**) Results of two independent QINESIn experiments where nucleosome stability was calculated by the loss of H2Av-GFP signal in nuclei isolated from *H2Av-GFP;+* (wild type) and *H2Av-GFP;HHH* (mutant) embryos. In both experiments normalized fluorescence intensity differs significantly at 800 mM NaCl treatment (nonparametric Mann-Whitney test; A: *P* = 0.0015; B: *P* = 0.0002). Error bar: SEM. (**C**) Combined results of experiments 1 and 2, showing the average values. In this case error bars represent the average SEM. (**D**) Percentage of chromatin-bound core histones (H3 and H4) after 800 mM NaCl salt elution, determined by Western blot. (**E**) Percentage of chromatin-bound linker histones prepared from *3F+HHH* mixed and *3F/HHH* heterozygous early embryonic chromatin, determined by Western blot. For statistics, two-tailed, unpaired t-test was used (D, E). (**F**) Percentage of chromatin-bound linker histones after 100–300 mM NaCl salt elution, prepared from *3F*+*HHH* mixed and *3F*/*HHH* heterozygous early embryonic chromatin, and determined by Western blot. Error bars represent SD (D, F).

Results of QINESIn assays showed that in nuclei obtained from embryos with linker histone BigH1 (wild type), nucleosomes were more resistant toward salt elution than nucleosomes containing H1 (*HHH*). The greatest difference between GFP signals in nuclei of *HHH* and wild type embryos was detectable at 800 mM sodium-chloride wash steps, which decreased chromatin associated signal more effectively in the *HHH* mutant than in the wild type (Figure 5A–C). The result of this assay thus indicates that although nucleosomal core histones are resistant to a rather high concentration of salt, GFP-tagged H2Av can be extracted more easily from nucleosomes formed in the presence of the canonical linker histone.

To test if other core histones H3 and H4 also show differences in salt elution, we isolated nuclei from early (1–2 h old) embryos, treated them with 800 mM sodium-chloride and analyzed the eluted and chromatin-bound proteins on western blot using H3 and H4 specific antibodies. We found that both H3 and H4 eluted from chromatin in greater ratio in the *HHH* mutant upon 800 mM salt treatment (Figure 5D and Supplementary Figure S5B). These data provide further support that nucleosomes formed in the presence of BigH1 linker histone are more stable.

We also assessed the resistance of BigH1 and H1 towards salt elution. Since the association of linker histones with DNA is weaker than that of core histones (29), in these experiments we applied salt washes of lower concentrations. As for H3 and H4, linker histone elution was analyzed by Western blot, taking advantage of the FLAG epitope attached to both H1 and BigH1 N-terminus. We performed these experiments using samples in which *3F* and *HHH* embryos were mixed in equal quantity (*3F*+*HHH*), and also with samples which were obtained from heterozygous embryos (*3F*/*HHH*). The use of these sample types permitted us to draw conclusions concerning both the competition and binding affinity of the two linker histones. Based on the results we could make two observations. First, BigH1 and H1 bind to chromatin in similar ratios in *3F+HHH* and *3F/HHH* samples, indicating no competition in nucleosome binding between the two linker histones (Figure 5E and Supplementary Figure S5A). Second, H1 binding to nucleosomes is more resistant to salt elution, since it eluted from chromatin in lesser quantity than BigH1 at all tested salt concentrations (Figure 5F and Supplementary Figure S5C). Combined, these data show that although nucleosomes formed in the presence of BigH1 are more stable, chromatosomes formed with BigH1 are less salt resistant. This indicates structural differences both in nucleosomes and chromatosomes formed in the presence of canonical somatic or germline and early embryo-specific linker histones.

## DISCUSSION

In *Drosophila*, an alternative linker histone BigH1 is present in the early embryo and in the male and female germline (12,30). To study the specific developmental role of BigH1, we generated transgenic animals that express chimera linker histones constructed by domain switches between somatic H1 and germline/early embryo-specific BigH1. Analysis of the resulting flies revealed that under normal conditions H1 can replace BigH1 effectively and the embryogenesis proceeds without major defects, which confirms the findings of recent studies (31,32). However, sublethal phenotypes, such as nuclear fallout can be observed in mutants where the central globular and C-terminal domains of BigH1 are replaced with those of H1, indicating that these protein regions are necessary for function. In embryos with these hybrid proteins, a large number of nuclei are removed from the cortical region, which can trigger an extra nuclear cycle. The fact that low density of nuclei causes extra precellular cleavage has also been observed by others (33). While mitotic defects occur more frequently in the absence of BigH1 than in wild type, at normal temperature the robust developmental program can compensate for this. However, at suboptimal temperature, development cannot proceed with H1 as the sole linker histone present. Comparison of the hatching rates of different H1/BigH1 hybrid carriers at low temperature also supports the hypothesis that the central and C-terminal domains of BigH1 are essential for its proper function, as in the absence of these animals show a wide range of defects during the early stages of nuclear divisions. These defects, most probably arising from failures in replication, could be so frequent that only a few groups of nuclei remain observable at later stages. As HBB hybrid carriers do not exhibit either nuclear fallout or cold sensitive phenotype despite HBB protein being less abundant in embryos, the observed defects are clearly not the result of low protein level.

A comparison of histone mobility by FRAP indicates that in early embryos which have H1 linker histone (*HHH*) the recovery of H2Av-GFP signal is decreased, particularly at the early S-phase, suggesting that H1 is less mobile than BigH1. Higher mobility has also been described for *Xenopus* and mice oocyte-specific linker histone variants (34,35). A higher density of lipid droplets containing core histones can also be detected in embryos in which H1 replaces BigH1, possibly as a consequence of less dynamic nucleosome exchange. This is in accord with the observation that the majority of histone exchange happens in early S-phase, concomitant with replication. The fact that replication activity is higher in early S-phase is indicated by the more abundant PCNA level in early than in late S-phase nuclei of NC 13 *Drosophila* embryos (36,37). These observations can be interpreted as signs of delay in replication and concomitantly, reduced core histone exchange in the presence of H1, which can explain the frequent mitotic defects.

The type of linker histone affects the resistance of nucleosomes to salt elution, as core histones H2A, H3 and H4 could be extracted at lower salt concentrations if the linker histone was H1. Interestingly however, H1 itself can be eluted at higher salt wash from chromatin compared to BigH1, even though the nucleosomes formed in its presence are less stable.

**Figure 6.**
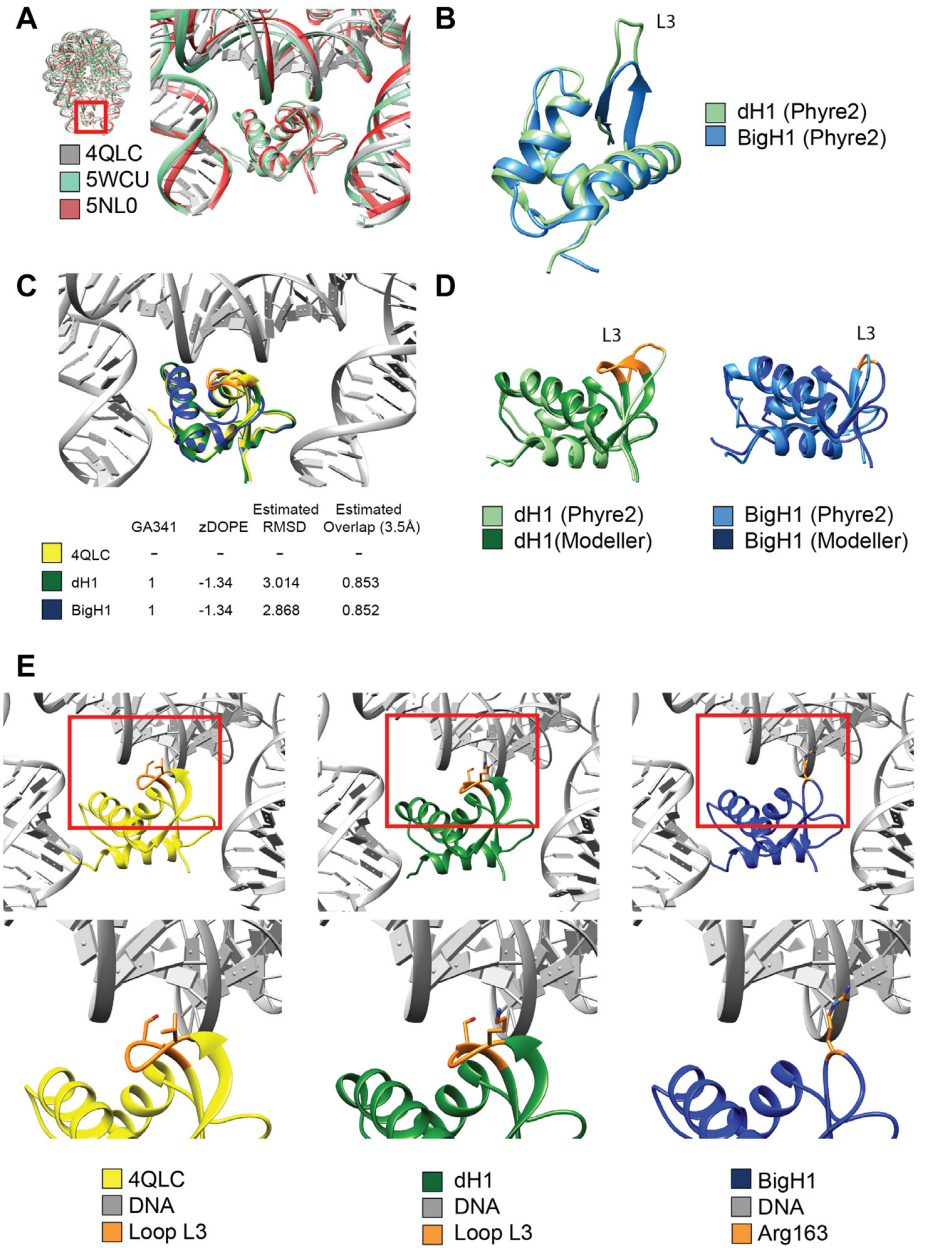
Comparison of H1 and BigH1 predicted globular domain structures reveals differences in presumed DNA binding loop. (**A**) Conserved 3D structure of the globular domains of Gallus (4QLC, 5WCU) and Xenopus (5NL0) linker histones in relation to nucleosomal DNA. (**B**) Structure predictions for dH1 and BigH1 indicates a shorter loop (L3) in BigH1. (**C**) Structural models for DNA-bound dH1 and BigH1 built on Gallus linker histone template (4QLC), with model quality indicators. (**D**) Comparison of the models obtained by the two indicated programs with highlighted alteration in the loop (L3) involved in DNA binding. (**E**) Enlarged structure of the loop (L3) involved in nucleosomal DNA binding. Two amino acids, Val87 and Ser90 (orange), described to be important in DNA binding by Zhou *et al.* (21) are missing from BigH1, suggesting difference between the two linker histones in DNA interactions.

All combined, based on the results of FRAP and nucleosome stability assays, we conclude that BigH1 is more mobile than H1 and binds nucleosomes less firmly; however, nucleosomes formed when BigH1 is the resident linker histone are more stable as the core histones are more strongly associated with DNA. To resolve the apparent contradiction, we propose that the binding of BigH1 to nucleosomes is weaker, allowing the histone octamer to slide more freely along the DNA and to engage with DNA segments where positioning is the most favored. On the other hand, H1 binds to nucleosomes more tightly. Thus, nucleosomes formed in the presence of somatic H1 are constrained into positions which are energetically less optimal, but if appropriate regulators are present this nucleosome arrangement can support the gene expression program. This can explain the conditional lethal phenotype of chimeras where the central globular and C-terminal domains of BigH1 (responsible for nucleosome binding) are replaced with those of H1. Since H1 establishes a stronger interaction with nucleosomes it does not permit their rapid disassembly–reassembly required for fast spaced nuclear divisions in the early embryo. This gives rise to several mitotic defects, possibly stemming from incomplete or faulty replication as indicated by nuclear fallout and the positive staining of descending nuclei both for phosphorylated γH2Av and PCNA. The difference in the effect of BigH1 and H1 is especially critical at low temperature, where the strong H1-nucleosome interaction essentially freezes nucleosomes in place; however, at 25°C with a less rigid interaction H1 is able to substitute for BigH1. The predicted difference between BigH1 and H1 in respect of their interaction with DNA (Figure 5) supports our hypothesis on differing chromatosome formation by H1 and BigH1. By modeling BigH1 structure based on the known structure of the chicken linker histone H5 we detected the lack of a loop including two amino acids that were described as crucial for binding nucleosomal DNA (21) (Figure 6).

We thereby suggest that BigH1 binds more preferably to the DNA entering/exiting and less to the DNA wound around the nucleosome, which would explain its higher mobility in FRAP and salt elution experiments. The altered binding can explain the observed minor difference in nucleosome spacing in the presence of H1 versus BigH1, indicating the latter more in contact with linker DNA. However, the average spacing of nucleosomes and nucleosome occupancy seem to be affected by the type of linker histone only very slightly as these features do not change greatly when BigH1 is replaced with H1. The details of nucleosome arrangements with H1 versus BigH1 can be revealed by genome-wide analyses of linker histone occupancy, such as that published recently for H1 in late embryonic chromatin (38).

A further interesting observation we made by investigating BigH1–H1 is that the two linker histones do not compete with each other for binding sites. This notion is supported by findings of Šatovic *et al.* who have found that in *in vitro* ChIP experiments H1 occupancy was not impaired upon BigH1 binding (39).

In summary, our data show that the higher mobility and relatively weaker nucleosome binding affinity of an oocyte specific linker histone, as is BigH1 in *Drosophila*, makes the alternative linker histone suitable for accommodating the fast paced nuclear divisions with rapid nucleosome exchange and assembly steps of early embryonic chromatin.

## DATA AVAILABILITY

CRISPR Optimal Target Finder (http://targetfinder.flycrispr.neuro.brown.edu/).

easyFRAP (https://easyfrap.vmnet.upatras.gr/?AspxAutoDetectCookieSupport=1).

UCSF Chimera (https://www.cgl.ucsf.edu/chimera/).

GraphPad Prism 6 (https://www.graphpad.com/scientific-software/prism/).

## Supplementary Material

gkaa624_Supplemental_Files
