# Supplementary material for: Alternative linker histone permits fast paced nuclear divisions in early Drosophila embryo
Source: Nucleic Acids Res. 2020 Jul 25;48(16):9007–18. doi: 10.1093/nar/gkaa624 (PMC7498357; doi:10.1093/nar/gkaa624)
Supplement: gkaa624_Supplemental_Files [file gkaa624_supplemental_files.zip › Supplementary file.pdf]

SUPPLEMENTARY DATA for Alternative linker histone permits fast paced nuclear divisions in early *Drosophila* embryo

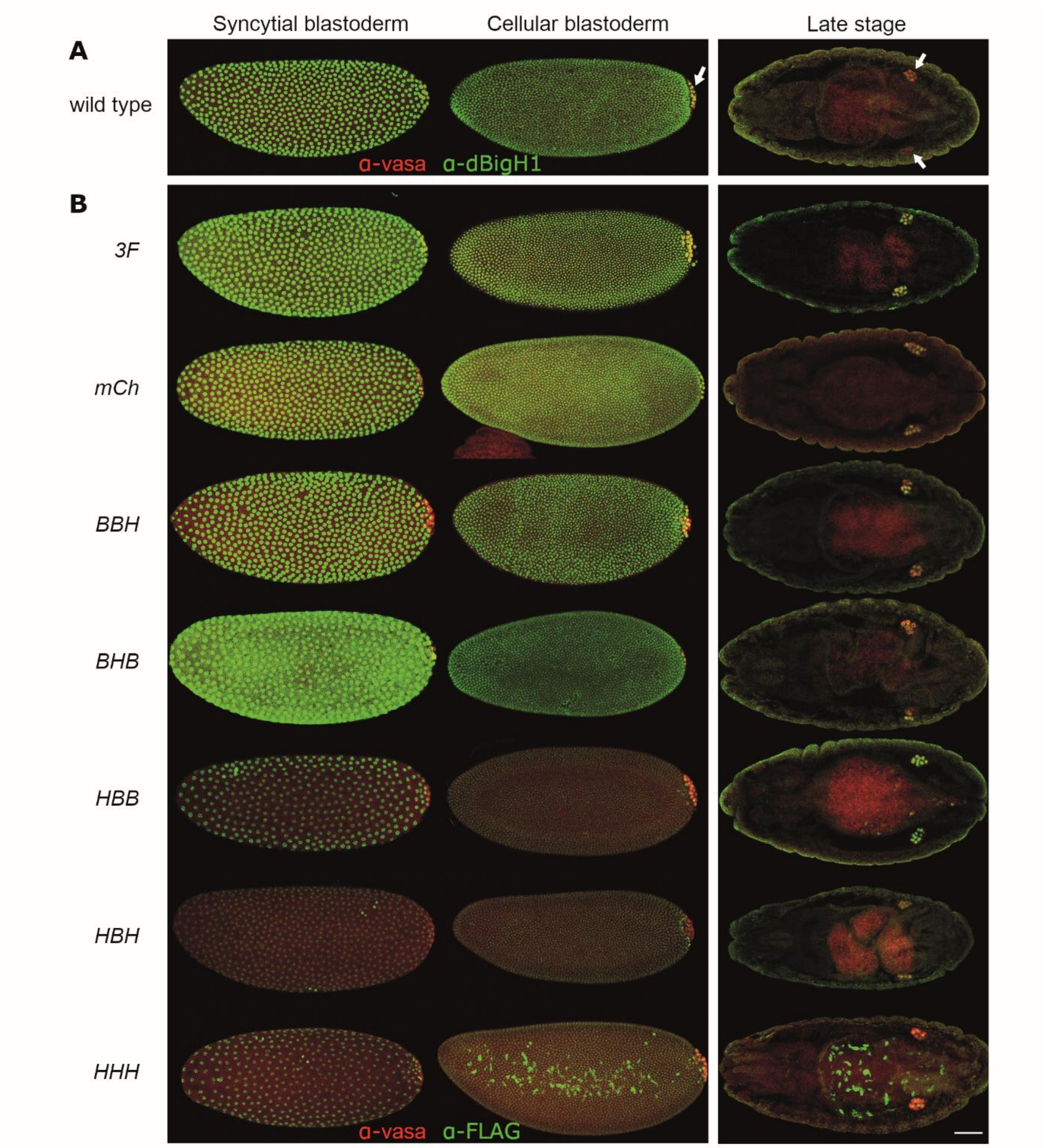

Figure S1. Expressional patterns of modified BigH1 proteins are similar to wild type.

Immunostaining of embryos in syncytial, cellular blastoderm and late stages. Primordial germ cells (marked with arrows in wild type) were stained with anti-vasa and nuclei were stained with anti-dBigH1 in wild type (A) and with anti-FLAG in mutant embryos (B). Confocal images of syncytial and cellular blastoderm embryos (left and

middle column) were made with the same microscope and image analysis settings, allowing the comparison of protein levels in mutants. Scale bar represents 50 µm.

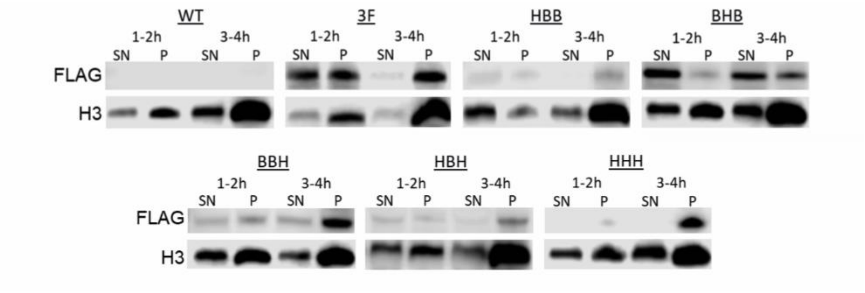

Figure S2. Protein expression levels are different in BigH1/H1 chimeras.

The level of chimeric proteins in 1-2 hour (syncytial blastoderm) and 3-4 hour (cellular blastoderm) old embryos compared to wild type FLAG-tagged BigH1 (3F), as determined by Western blot using anti-FLAG antibodies. Chromatin (pellet, P) and non-chromatin fractions (supernatant, SN) were analysed. Wild type animals where BigH1 is not tagged with FLAG were used as negative control. Histone H3 level was used as a loading control.

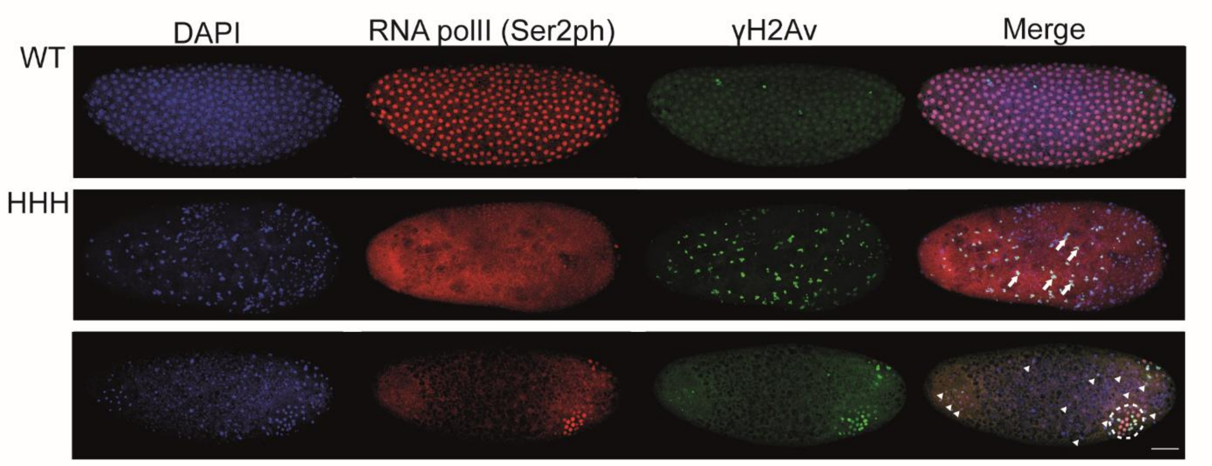

Figure S3. Phenotypes of *HHH* mutant embryos at 15 °C.

Immunostaining of wild type and mutant syncytial blastoderm embryos kept at 15°C after egg laying. DNA was stained with DAPI, elongating RNA polymerase with anti-RNA polII Ser2ph and double stranded-DNA breaks with anti-γH2Av. On the middle panel, arrows indicate aggregated DNA. On the bottom panel, nuclei islet showing

RNA polII Ser2ph staining is marked with dashed line, while other nuclei are RNA polII Ser2ph negative (arrowheads). Scale bar represents 50 µm.

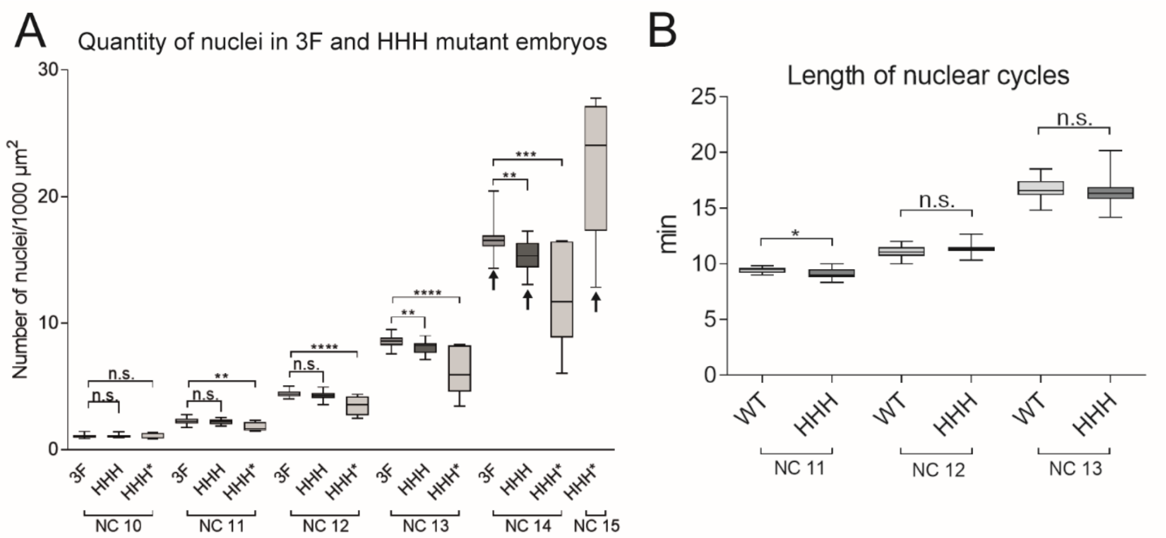

Figure S4. Nuclear quantity and nuclear cycle lengths in wild type and *HHH* mutant.

A) Wisker plot (min to max) visualization of number of nuclei/area in *3F* (corresponding to wild type) and *HHH* mutant embryos from NC10 to cellularization. *HHH* mutant embryos which show extra precellular nuclear cycle are marked with asterix. Arrows indicate the time of cellularization. B) Wisker plot (min to max) visualization of nuclear cycles lengths in wild type and *HHH* mutant embryos. 25-28 wild type and mutant embryos were analyzed. In NC 11, there is a slight difference between wild type and mutant, while in NC 12 and NC 13 differences are not significant. Error bars represent SD, p-values: *<0.05; **<0.01; ***<0.001; ****<0.0001.

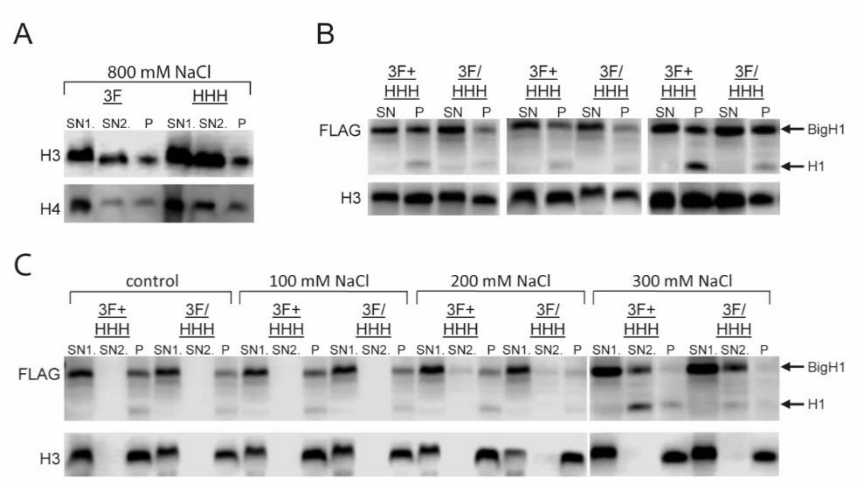

Figure S5. Western blot analysis of chromatin-bound and soluble histone proteins shows differences in wild type and HHH mutant embryos.

A) Representative Western blot of the salt elution (800 mM) of H3 and H4 core histones from *3F* and *HHH* embryonic chromatin. Statistics for three independent experiments are provided on Figure 5D. B) The level of BigH1 and H1 proteins in *3F*+*HHH* mixed and *3F*/*HHH* heterozygous embryos (1-2 h old). Statistics for these experiments are provided on Figure 5E. C) Representative Western blot of the salt elution of linker histones (BigH1, H1) from *3F*+*HHH* mixed and *3F/HHH* heterozygous embryonic chromatin. SN1.: non-chromatin-bound fraction, SN2.: salt eluted fraction, P: chromatin-bound fraction after salt elution. Relevant bands are grouped together and are part of the same experiment. The combined result for three independent experiments is provided on Figure 5F.

Primary antibodies	Host species, clonality	Dilution	Source	Showed in	Application
anti-γH2Av (UNC93)	mouse monoclonal	1:500	DSHB	Figure 3; Figure S2.	IHC
anti-BigH1	rat polyclonal	1:600	Azorin lab	Figure S1.	IHC
anti-FLAG M2 (F1804)	mouse monoclonal	1:1000, 1:2000	Sigma	Figure S2.	IHC, WB
anti-RNApolII Ser2ph, (ab5095)	rabbit polyclonal	1:400	Abcam	Figure S2.	IHC
Anti-PCNA (sc-56)	mouse monoclonal	1:500	SCBT	Figure 2.	IHC
anti-H3 (ab1791)	rabbit polyclonal	1:5000	Abcam	Figure S2.	WB
anti-H4 (ab10158)	rabbit polyclonal	1:1000	Abcam	Figure 5., Figure S5.	WB
**Secondary antibodies**					
Alexa 488 conjugated anti-mouse (A11001)	goat polyclonal	1:600	Invitrogen	Figure 3; Figure S1-2.	IHC
Alexa 546 conjugated anti-rat (A11081)	goat polyclonal	1:600	Invitrogen	Figure S1.	IHC
Alexa 546 conjugated anti-rabbit (A11010)	goat polyclonal	1:600	Invitrogen	Figure S2.	IHC
Goat Anti-Rabbit Immunoglobulins/HRP	goat polyclonal	1:10000	Dako	Figure S2., Figure 5., Figure S5.	WB
Rabbit Anti-Mouse Immunoglobulins/HRP	rabbit polyclonal	1:10000	Dako	Figure S2., Figure 5., Figure S5.	WB

Table S1. Primary and secondary antibodies used in immunostaining.

Movie S1. mCherry tagged BigH1 shows colocalization with GFP tagged H2Av proteins during nuclear cycle of syncytial blastoderm Drosophila embryo.

In vivo videomicroscopy represents the localization mCherry tagged BigH1 and GFP tagged H2Av histones from NC 12 prophase (late S-phase) to NC 13 prometaphase. On merged video BigH1-mCherry is red, H2Av-GFP is green. Time resolution is 20 frame/min. Scale bar represents 5 μm.

Movie S2. Extra precellular cleavage in *HHH* mutant embryos.

In *H2Av-GFP;* + (wild type) syncytial blastoderm embryo (upper video) four round of nuclear cycles (NC 10-13) come before cellularization while in *H2Av-GFP; HHH* mutant embryo (lower video) an extra nuclear cycle can be observed resulting five rounds of precellular cycles (NC 10-14). This phenotype was observed in 5 of 24 embryos. Time resolution is 6 frame/min, scale bar is 50 μm.
